# Diversification and deleterious role of microbiome in gastric cancer

**DOI:** 10.1002/cnr2.1878

**Published:** 2023-08-02

**Authors:** Indranil Chattopadhyay, Rohit Gundamaraju, Ashwin Rajeev

**Affiliations:** ^1^ Department of Biotechnology Central University of Tamil Nadu Thiruvarur India; ^2^ ER stress and Mucosal Immunology Team School of Health Sciences, University of Tasmania Launceston Tasmania Australia

**Keywords:** bacterial metabolites, gastric cancer, gut microbiome, *Helicobacter pylori*

## Abstract

Gut microbiota dictates the fate of several diseases, including cancer. Most gastric cancers (GC) belong to gastric adenocarcinomas (GAC). *Helicobacter pylori* colonizes the gastric epithelium and is the causative agent of 75% of all stomach malignancies globally. This bacterium has several virulence factors, including cytotoxin‐associated gene A (CagA), vacuolating cytotoxin (VacA), and outer membrane proteins (OMPs), all of which have been linked to the development of gastric cancer. In addition, bacteria such as *Escherichia coli*, *Streptococcus*, *Clostridium*, *Haemophilus*, *Veillonella*, *Staphylococcus*, and *Lactobacillus* play an important role in the development of gastric cancer. Besides, lactic acid bacteria (LAB) such as *Bifidobacterium*, *Lactobacillus*, *Lactococcus*, and *Streptococcus* were found in greater abundance in GAC patients. To identify potential diagnostic and therapeutic interventions for GC, it is essential to understand the mechanistic role of *H. pylori* and other bacteria that contribute to gastric carcinogenesis. Furthermore, understanding bacteria‐host interactions and bacteria‐induced inflammatory pathways in the host is critical for developing treatment targets for gastric cancer.

## 1 INTRODUCTION

The microbiome of the gastrointestinal tract consists of the total genomic content of bacteria, viruses, and archaea. The gut microbiota is involved in the host's immune response and metabolism. Dysbiosis of the gut microbiome induces tumorigenesis along with other confounding factors such as diet, tobacco and alcohol consumption, and other environmental factors. Gastric cancer is a classic example of the interaction between dysbiosis of the gut microbiota and epithelial cells of the gut of the host.^1^ Gastric cancer is the fourth most common cancer in the world.^2^ Gastric cancer is most common in China, Japan, southern and Eastern Europe, as well as South and Central America.^3^ The majority of gastric cancers (GC) are gastric adenocarcinomas (GAC). As per Lauren's classification, GC is histologically classified into intestinal, diffuse, mixed and non‐classifiable.^4^ According to World Health Organization (WHO) criteria, GAC is classified as papillary, mucinous, tubular, and signet ring cell.^5^ The Cancer Genome Atlas (TCGA) classified GC into four major subtypes such as Epstein–Barr virus (EBV)‐positive, microsatellite instability (MSI), genomically stable (GS), and chromosomal instability (CIN).^6^ Sequential events such as superficial gastritis, chronic atrophic gastritis, intestinal metaplasia, and dysplasia are involved in the development of GAC.^7^ GAC is a multifactorial inflammatory disorder. Tobacco smoking, alcohol consumption, high salt and smoked meat consumption, low consumption of vegetables and fruits, iron deficiency, obesity, microbial infections, and host genetic factors are major risk factors for GAC.^8^, ^9^ *H. pylori* contribute significantly to the early stages of GAC. However, other gastric microbiotas are also involved in the development of GAC.^8^ *Helicobacter pylori*, which colonizes the gastric epithelium, is responsible for 75% of all gastric cancers worldwide.^10^ Other than *H. pylori*, the stomach harbors to nearly 10^3^–10^4^ bacteria.^10^ Wang Z. et al., 2020 reported that the diversity of bacteria was gradually reduced in normal healthy individuals, non‐atrophic chronic gastritis, intestinal metaplasia, and gastric cancer.^11^ *H. pylori* induce the progression of the gastric mucosa to develop gastric cancer through sequential alterations of the gastric mucosa such as atrophic gastritis (AG), intestinal metaplasia (IM), and dysplasia.^12^ The oncoprotein CagA and vacuolating cytotoxin A (VacA) of *H. pylori* are responsible for infection.^13^ CagA induces tumorigenesis through activation of cell proliferation, inflammation, loss of contact inhibition, and suppression of apoptosis. VacA is involved in immunosuppression through the prevention of regulatory T cells activity, activation of mast cells to secrete proinflammatory cytokines, and cell differentiation through activation of Wnt/beta‐catenin and the MAP kinase pathway.^14^ *H. pylori* induce up‐regulation of TLR9, which enhances the risk of GC development.^15^ *Escherichia coli*, *Streptococcus*, *Clostridium*, *Haemophilus*, *Veillonella*, *Staphylococcus*, *Neisseria*, *Nitrospirae*, and *Lactobacillus* showed a significant contribution to the development of gastric cancer through the production of carcinogenic N‐nitroso compounds.^16^ Lactic acid bacteria (LAB) are responsible for the production of reactive oxygen species (ROS), which induce DNA damage and reduce nitrate to nitrite which drive activation of the oncogenes, enhance angiogenesis, and inhibit apoptosis.^8^ *Clostridium sporogenes*, *Ruminococcus gnavus*, and *Lactobacillus* sp. are involved in the progression of the tumor through the inhibition of antitumor immune responses.^9^ Metabolites from the gut microbiota influence the development of tumorigenesis as well as improve anticancer therapy in GC patients.^17^ Interactions between *H. pylori* and other bacteria could possibly play a role in the development of gastric cancer. Gut microbiota must play an important target for next‐generation cancer therapy. This study also indicates the potential application of pharmacomicrobiomics in the treatment of gastric cancer. The present narrative review provides a bird's eye view of the role of alterations in gut microbiota and their metabolites in gastric cancer.

## 2 BACTERIAL DIVERSITY IN STOMACH OF HEALTHY INDIVIDUALS AND GASTRIC CANCER PATIENTS

The microbial load varies in the different parts of the gastrointestinal tract, such as 10^12^ CFU/mL (oral cavity), 10^7^ CFU/mL (stomach), and 10^14^ CFU/mL (colon). Due to the lower oxygen concentration in the upper gastrointestinal tract, Gram‐positive cocci such as *Gemella* and *Streptococcus* are predominant, whereas *Clostridium* and *Faecalibacterium* are predominant in the intestines and colon. *Akkermansia muciniphila*, *Bacteroides thetaiotaomicron*, *Bacteroides fragilis*, *Bifidobacterium bifidium*, and *Ruminoccous gnavus* are involved in the utilization of glycans of the mucus layer of the gastric region through the action of enzymes such as glycosidase, sulphatase, and sialidase.^18^ Age, diet, ethnicity, sex, lifestyle, use of antibiotics and proton pump inhibitors (PPI), and infection with *H. pylori* may modulate alterations in the gut microbiome.^19^ Bacteria and fungus are unable to survive in the stomach due to its low pH. Metagenomic analysis of mucosal biopsies of healthy subjects revealed that phylum Proteobacteria is the most predominant, followed by Firmicutes, Bacteroidetes, Actinobacteria, and Fusobacteria. *Actinobacillus*, *Bacillus*, *Corynebacterium* spp., *Haemophilus*, *Neisseria*, *Prevotella*, *Pseudomonas*, *Propionibacterium*, *Lactobacillus*, *Lactococcus*, *Staphylococcus*, *Streptococcus*, *Stenotrophomonas*, and *Veillonella* are the predominant bacterial genera in the gastric region of healthy individuals.^20^, ^21^, ^22^, ^23^ *Haemophilus*, *Lactobacillus*, *Prevotella*, *Streptococci*, *Veillonella*, and *Neisseria* showed higher abundance in the gastric mucosa of gastric cancer patients.^23^ Firmicutes are mainly represented by *Clostridium*, *Dorea*, *Eubacterium*, *Ruminococcus*, *Peptostreptococcus*, and *Lactobacillus*.^24^ *Cladosporium*, *Candida*, and *Saccharomyces* are common fungal genera found in the human gut.^25^ Anti‐tumor immune responses are activated by gut bacteria via T‐cell activation against bacterial virulence factors.^26^

*Lactobacillus coleohominis* and *Lachnospiracea* showed higher abundance, but *Porphyromonas* and *Neisseria* showed lower abundance in gastric cancer patients. *Pseudomonas* showed a significantly higher abundance in gastric cancer.^27^ *Clostridium* and *Prevotella* showed higher abundance in the mucosa of gastric cancer patients.^21^ The relative abundance of *Clostridium*, *Fusobacterium*, *Lactobacillus*, *Lachnospiraceae*, *Leptotrichia*, *Streptococcus*, *Veillonella*, *and Prevotella* was increased in gastric cancer patients.^23^ *Fusobacterium*, *Streptococcus*, *Prevotella*, and *Leptotrichia* showed higher abundance in intestinal metaplasia.^28^

Dietary patterns, lifestyle, age, gender, and geographical variation might influence gut microbiota alterations.^29^ *Achromobacter*, *Clostridium*, *Citrobacter*, and *Rhodococcus* showed higher abundance in gastric cancer patients from Portuguese populations.^30^ *Lactobacillus* showed higher abundance during the progression of gastric cancer patients in the Mexican, Swedish, and Taiwanese populations.^14^, ^23^ In gastric tumors, *Prevotella copri* and *Bacteroides uniformis* were found in lower abundance, while *Propionibacterium acnes*, *Prevotella melaninogenica*, and *Streptococcus anginosus* were found in higher abundance as compared to healthy individuals.^31^ *P. acnes* induce the progression of lymphocytic gastritis to develop GC thorough the secretion of IL‐15. *P. copri* induce secretion of redox protein, which is responsible for the development of inflammation in GC.^32^ *Anoxybacillus*, *Novosphingobium*, *Ochrobactrum*, *Pseudoxanthomonas* and *Ralstonia* showed higher abundance in the early stage of gastric cancer patients, whereas *Burkholderia*, *Salinivibrio*, *Tsukamurella*, and *Uruburuella* enriched in the advanced stage of gastric cancer patients.^11^ Oral bacteria such as *Dialister pneumosintes*, *Fusobacterium*, *Peptostreptococcus stomatis*, *Parvimonas micra*, *S. anginosus*, and *Slackia exigua* showed higher abundance in gastric cancer patients. Changes in stomach pH may cause oral bacteria to aggregate together in the gastric area.^33^ Bacterial genera such as *Clostridium*, Fusobacterium, *Lactobacillus*, *Lachnospiraceae*, *Leptotrichia*, *Prevotella*, *Streptococcus*, and *Veillonella* showed higher abundance in GAC patients.^34^ The presence of *Fusobacterium nucleatum* showed an association with a worse prognosis in Lauren's diffuse‐type GC and the expression of p53 in tumor tissue.^35^, ^36^

**FIGURE 1:**
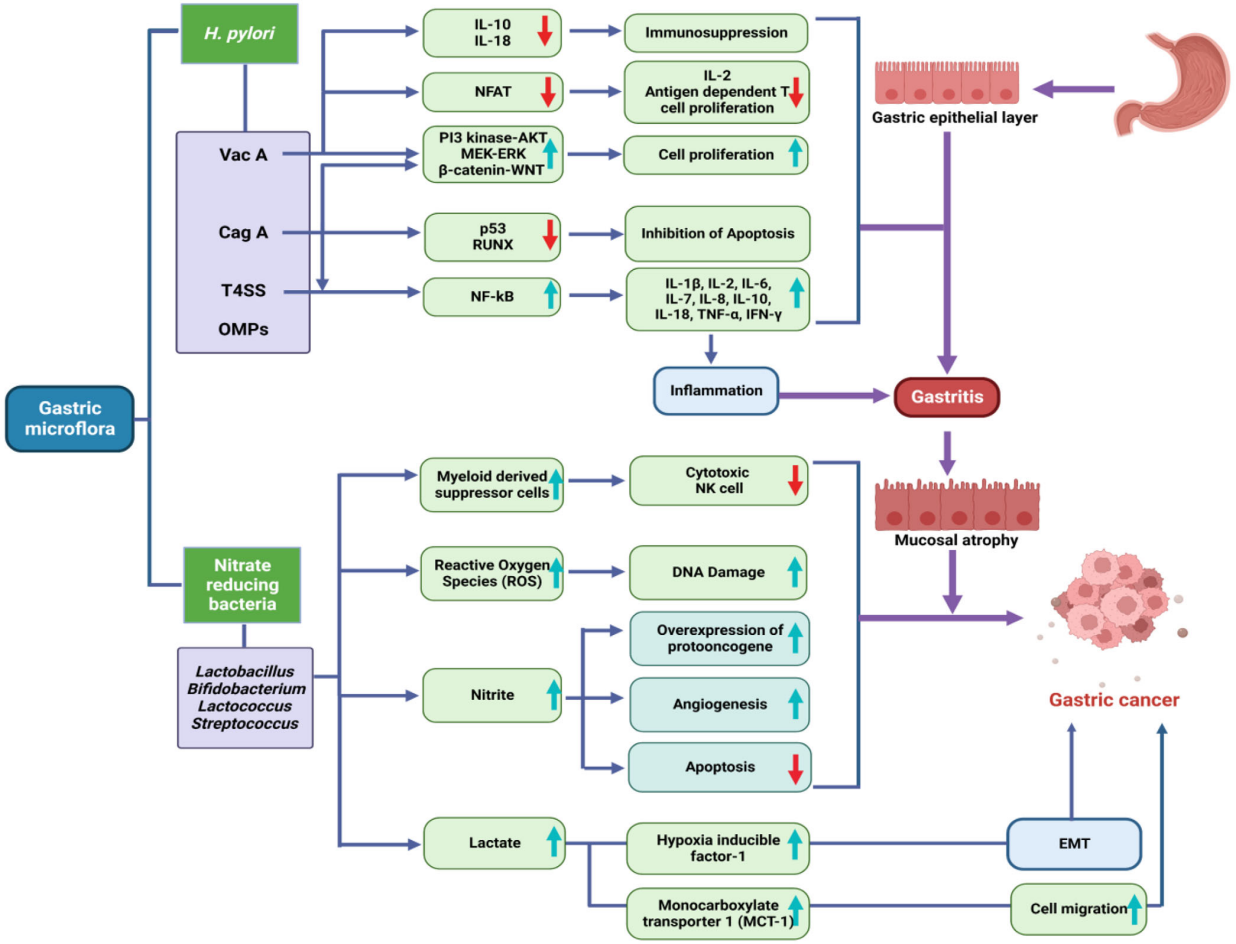
Role of virulent factors of *H. pylori* and other bacteria in the development of gastric cancer through alteration of hallmarks of tumorigenesis. The figure illustrates the mechanistic role of *H. pylori* and *Lactobacillus* in progression of gastric cancers. *H. pylori* activate several pro‐inflammatory pathways and cytokines which in turn trigger pre‐carcinogenic switches. Likewise, *Lactobacillus* is responsible for triggering of ROS, DNA damage agents and other drivers responsible for initiating gastric cancer.

Lactic acid bacteria (LAB) such as *Bifidobacterium*, *Lactobacillus*, *Lactococcus*, and *Streptococcus* showed significantly higher abundance in patients with gastric adenocarcinoma (GAC).^8^ Colonization of LAB in gastric atrophic mucosa induces the over growth of oncobacteria such as *Veillonella*, *Prevotella*, *Fusobacterium*, and *Leptotrichia* in patients with GAC. LAB is responsible for the production of reactive oxygen species (ROS), which induce DNA damage. LAB is involved in the reduction of nitrate to nitrite, which drives mutagenesis, over expression of the proto‐oncogene, enhancement of angiogenesis, and inhibition of programmed cell death^8^ (Figure 1). LAB is also involved in epithelial mesenchymal transition.^37^ The levels of L‐lactate, D‐lactate, and D‐LDH showed higher abundance in GAC patients.^38^ *H. pylori* induce overexpression of DLDH in atrophic gastritis patients.^39^ LAB induces colonization of *non‐H. pylori* carcinogenic bacteria.^84^ LAB enhances the secretion of exogenous lactate which induces cell migration through the activation of monocarboxylate transporter 1 (MCT1) and epithelial mesenchymal transition through the activation of hypoxia‐inducible factor‐1 (HIF‐1). Lactate develops the chemoresistance property of tumors through the expression of hydrocarboxylic acid receptor 1 (HCAR1) and MCT1 (Figure 1). Lactate induces the expression of VEGF and also drives overexpression of myeloid derived suppressor cells, which prevent the cytotoxic activity of natural killer cells.^28^ *Bifidobacteria* induces over expression of type I interferon (IFN) in antigen‐presenting cells of secondary lymphoid organs.^40^ LAB may have an effect on GAC by inducing secretions of lactate, ROS, and N‐nitroso compounds, as well as EMT.

## 3 ROLE OF *HELICOBACTER PYLORI* IN GASTRIC CANCER

The pH of the gastric region varies from pH 1–2 in the gastric lumen region and pH 6–7 on the mucosal surface.^41^ Microorganisms prefer growing on the mucosal surface.^42^ Bacteria enter the stomach from the upper digestive tract and respiratory tract.^43^ *Helicobacter pylori*, which colonizes in the acidic environment of the stomach, are responsible for the development of noncardiac adenocarcinomas. It is a Gram‐negative, spiral‐shaped, flagellated bacterium that belongs to the phylum Proteobacteria.^16^ It possesses urease, catalase, and oxidase activities.^19^ This bacterium is responsible for the development of atrophic gastritis, chronic gastritis, gastric adenocarcinoma, and mucosa associated lymphoid tissue lymphoma (MALT).^44^

Blood group antigen‐binding adhesion (BabA), sialic acid binding adhesion protein (SabA), AlpA/B, HopZ, and OipA are outer membrane proteins of *H. pylori* that bind to the gastric epithelium. *H. pylori* induce gastric epithelial cells to express sialyl‐dimeric‐Lewis glycosphingolipid, which acts as a receptor for SabA.^45^ Adhesin BabA of *H. pylori* binds with ABO/Leb blood‐group antigens on gastric epithelial cells.^46^ This bacterium has several virulence factors, including cytotoxin‐associated gene A (CagA), vacuolating cytotoxin (VacA), and outer membrane proteins (OMPs), all of which have been linked to the development of gastric cancer^47^ (Figure 1). This bacterium induces the development of gastric cancer through the damage of hydrochloric acid‐secreting gastric glands.^48^

CagA protein has a molecular weight of 120 to 140 kD and is secreted in the cytosol of the bacteria. The genes that encode CagA and Cag type IV secretion system (T4SS) are located in cag Pathogenicity Island (cag PAI). The CagA protein of *H. pylori* enters the host cell through the cag pathogenicity island (cagPAI)‐encoded type IV secretion system (T4SS). CagA induces cell proliferation by activating PI3 kinase–AKT, MEK–ERK, and β‐catenin–WNT pathways. CagA prevents apoptosis of gastric epithelial cells through the inhibition of p53 and RUNX. CagA and T4SS induce gastric carcinoma by activating NF‐κB pathways that drive inflammation and reactive oxygen species mediated DNA damage.^49^ CagA is involved in chronic gastritis; mucosa associated lymphoid tissue lymphoma, and gastric cancer in humans.^5^, ^50^ CagA of *H. pylori* is involved in the epigenetic silencing of miRNA let‐7 which drives overexpression of Ras in gastric carcinogenesis.^51^ CagA is phosphorylated by Src kinases at the Glu‐Pro‐Ile‐Tyr‐Ala (EPIYA) motifs in gastric epithelial cells. This event triggers the activation of pathways such as JAK/STAT3, NF‐κB, PI3K/Akt, Wnt/β‐catenin, and Ras/Erk pathways that drive carcinogenesis.^52^ CagA also induces activation of tyrosine phosphatase SHP2 through binding with SRC homology 2 domains (SH2), which drives the malignant transformation of gastric epithelial cells.^53^ CagA disrupts adherens junctions (AJs) in gastric epithelial cells.^54^ CagA triggers epithelial‐mesenchymal transition (EMT) in gastric cells.^55^

*H. Pylori* has urease activities, which are responsible for the enhancement of pH in the gastric region through the transformation of urea into ammonia and bicarbonate. The flagellum of *H. pylori* is responsible for the invasion of the gastric mucosa. The adhesions (HopQ, HopP, and HopS) of *H. Pylori* are responsible for *H. pylori* adhesion to gastric epithelial cells. The virulence factors CagA and VacA of *H. pylori* are responsible for the cytotoxic effect.^56^ Genetic alterations in Cag A and VacA of *H. pylori* are responsible for the enhancement of inflammation through the secretion of IL‐22 and IL‐8, the infiltration of neutrophils, and the colonization of bacteria through their outer membrane proteins such as BabA, and HopH.^57^ VacA is also involved in the over‐expression of VEGF, MAP kinase, and ERK1/2, and the activation of the Wnt/β‐catenin signaling pathway, and PI3K/Akt signaling pathway which drive proliferation and differentiation of gastric epithelial cells.^58^ VacA inhibits the dephosphorylation of the transcription factor nuclear factor of activated T cells (NFAT), which blocks the transcriptional activation of IL‐2 and antigen‐dependent cell proliferation.^59^ VacA exhibits immunosuppression through the activation of secretion of anti‐inflammatory cytokines such as IL‐10 and IL‐18 from dendritic cells that drive differentiation of Treg cells.^59^ Due to the immunosuppressive property of VacA, *H. pylori* may bypass the host immune system, which drives the survival of gastric tumors.^60^ The outer inflammatory protein (OipA) of *H. pylori* induces ulcers in the duodenum. CagL induces hypergastrinemia, which drives the development of gastric adenocarcinoma. CagL interacts with α5β1 integrin, which causes the host cell to secrete IL‐8.^61^

*H. pylori* induces inflammation in gastric epithelial cells through activation of NF‐κB which drives the secretion of inflammatory cytokines such as interleukin‐1β (IL‐1b), IL‐2, IL‐6, IL‐7, IL‐8, IL‐10, IL‐18, interferon‐γ, and TNF‐α.^62^ *H. pylori* also induce inflammation by activating Cyclooxygenase‐2 (COX‐2) and prostaglandin PGE2.^62^ Pathogen‐associated molecular patterns (PAMPs) of *H. pylori* bind with pattern recognition receptors (PRR) on host cells, which induce innate immune responses. TLRs of PRR also bind with flagellin, LPS, lipoteichoic acid, and lipoproteins of *H. pylori*. These events induce the secretion of inflammatory cytokines such as IL‐1, IL‐2, IL‐6, IL‐8, IL‐12, TNF‐α, and IFN‐γ.^24^

*H. pylori* are also involved in the epigenetic regulation of gastric epithelial cells. This bacterium is involved in the DNA methylation of CpG islands of E‐cadherin and tumor‐suppressor genes such as trefoil factor 2 (TFF2) and a forkhead box transcriptional regulator (FOXD3), which drives the development of gastric cancer.^63^, ^64^ *H. Pylori* induces hypermethylation of the promoter region of the RUNX3 tumor suppressor gene in gastric epithelial cells.^65^ This bacterium is involved in the methylation of the promoter region of miR‐210, which induces the proliferation of gastric cells through over expression of STMN1 and DIMT1.^66^ *H. pylori*‐positive gastric cancer patients showed a higher degree of promoter hypermethylation of tumor suppressor genes such as CDKN2A, APC, and p41ARC. *H. pylori* infection induces the hypermethylation of Connexin 32 (Cx32) and Connexin 43 (Cx43) in gastric cancer patients. It also induces the hypermethylation of O6‐methylguanine DNA methyltransferase (MGMT) in the gastric cancer patients.^67^

*H. pylori* prompt the expression of cytidine deaminase, which induces double‐strand breaks in gastric epithelial cells.^68^ *H. pylori* activate epidermal growth factor receptor (EGFR) through PI3K/AKT pathway.^69^ *H. pylori* aid in the production of reactive oxygen and nitrogen species (ROS/NOS) from neutrophils, macrophages, and vascular endothelial cells, which induce DNA damage and activate apoptosis or autophagy in gastric epithelial cells. ROS activates tyrosine phosphorylation at the C terminus of SHP2.^70^, ^71^ Bacterial peptidoglycans also activate the nucleotide‐binding oligomerization domain (NOD). Outer membrane vesicles of gram‐negative bacteria bind with NOD‐2, whereas the virulent factor of *H. pylori* binds with NOD‐1.^16^ *H. pylori* infection reduces the level of vitamin B12 in the serum, which enhances the risk of developing non‐cardia gastric adenocarcinoma.^9^ *H. pylori* inhibit the expression of miR‐22 and NLR family pyrin domain containing 3 (NLRP3), which drives the progression of GC through over expression of cyclin D1.^72^ TLR1 rs4833095 and TLR10 rs10004195 polymorphisms contribute significantly to the infection of *H. pylori* to gastric epithelial cells.^73^ *H. pylori* induced the expression of PD‐L1 in gastric epithelial cells and prevented the proliferation of CD4+ T cells in the blood.^26^ CD4+ T cells showed higher expression in gastric cancer, whereas CD8+ T cells showed lower expression.^74^

MALT lymphoma of the stomach is a slow growing B‐cell neoplasia. *H. pylori* infection is primarily responsible for the development of gastric MALT lymphomas.^75^ Chronic inflammation induces the development of mucosal associated lymphoid tissues (MALT) in the gastric mucosa.^76^ The CagA protein of *H. pylori* contributes significantly to the development of gastric MALT lymphoma through inhibition of B cell apoptosis and proliferation of B cells.^76^ *Streptococcus bovis*, *Achromobacter xylosoxidans*, and *Haemophilus influenzae* are also responsible for the development of gastric MALT lymphomas.^77^

Prolonged *H. pylori* infection causes an inflammatory response, which leads to atrophic gastritis and an increased risk of developing GC. The CagA protein induces cell proliferation, blocks apoptosis, and interrupts cell–cell adhesions, which drive the development of gastric tumors.

## 4 DIVERSITY MICROBIOTA IN GASTRIC REGION OF PATIENTS WITH GASTRIC CANCER IN THE PRESENCE AND ABSENCE OF *H. PYLORI*

Inflammation elevates the pH of the stomach, reducing the abundance of *H. pylori* while increasing the abundance of non‐*H. pylori* bacteria in the stomach.^78^ *H. pylori* induce inflammation in the gastric region, whereas other bacteria are responsible for the enhancement of inflammation, development of dysplasia, and the progression of gastric adenocarcinoma.^79^ *Helicobacter pylori* are responsible for the modification of the mucous layer of the gastric region which drives dysbiosis of gastric microbiota. In *H. pylori* positive individuals, Actinobacteria, Bacteroidetes, Firmicutes, and Proteobacteria showed higher abundance at the phylum level and *Streptococcus* showed higher abundance at the genus level.^56^ *Pseudomonas* and *Staphylococcus* are common bacterial genera in *H. pylori* free children's stomach.^41^ A higher abundance of Acidobacteria, Proteobacteria, and Spirochetes have been reported in *H. pylori* positive gastric cancer patients compared to *H. pylori* negative gastric cancer patients.^80^ γ‐Proteobacteria, β‐Proteobacteria, Bacteroidia, Flavobacteria, Fusobacteria or Negativicutes and *Clostridia* showed higher abundance in *H. pylori*‐negative gastric cancer patients.^41^ A higher abundance of Firmicutes and Bacteroidetes and lower abundance of Proteobacteria and Actinobacteria were reported in gastric cancer patients after gastrectomy.^81^ Distal gastrectomy induced the abundance of *Escherichia/Shigella*, *Veillonella*, and *Clostridium XVIII* and reduced the abundance of *Bacteroides*.^82^ *Prevotella copri* showed significantly higher abundance in *H. pylori*‐positive patients.^83^ Patients with atrophic gastritis showed higher abundance of *Lactobacillus* and *H. Pylori*.^84^ Fusobacteriaceae, Helicobacteraceae, Prevotellaceae, and Streptococcaceae are the predominant taxa in patients having chronic atrophic gastritis. Metabolic enzymes such as alanine dehydrogenase, glycolate oxidase, fumarate reductase, and ketol‐acid reductor isomerase are significantly elevated whereas succinate dehydrogenase is significantly reduced in *H. pylori*‐induced atrophic gastritis.^85^ The abundance of *Tannerella*, *Treponema*, and *Prevotella* spp. was significantly reduced in atrophic gastritis patients.^86^ Higher abundance of *Coriobacteriaceae*, *Enterococcaceae*, *Succinivibrio*, and *Rikenellaceae* was reported in individuals having *H. pylori* infection.^87^ *H. pylori* infected individuals showed an elevation of metabolic pathways related to fatty acid metabolism, xenobiotics metabolism by cytochrome P450, glycosphingolipid biosynthesis, N‐glycan biosynthesis, glycosaminoglycan degradation, and LPS biosynthesis. Peptidoglycan biosynthesis was reduced in individuals with *H. pylori* infection.^88^ *H. pylori* may provide a favorable environment for the development of opportunistic pathogens that enhance the progression of GC.

## 5 ROLE OF BACTERIAL METABOLITES IN GASTRIC CANCER

Bile acids (BAs), branched‐chain amino acids (BCAAs), short‐chain fatty acids (SCFAs), trimethylamine N‐oxide (TMAO), tryptophan, and indole derivatives are derived from gut microbiota.^89^ Free BAs, such as cholic acid, deoxycholic acid, and chenodeoxycholic acid induce apoptosis and inhibit the secretion of interleukin 6 (IL‐6) whereas conjugated BAs such as glycolic acid, glycodeoxycholic acid, and glycochenodeoxycholic acid induce cell proliferation and inflammation through secretion of IL‐6.^90^ SCFAs produced by gut bacteria are implicated in T cells modulation through the expression of G‐protein‐coupled receptors GPR41 or GPR43.^91^ The gut microbiome regulates host metabolism and immune response through the synthesis of spermidine and Vitamin B6.^92^ Gut bacteria‐released metabolites promote the progression and development of gastric cancer. SCFAs, polyamines, and metabolites derived from tryptophan catabolism contribute significantly to the development and progression of the tumor through alterations of epigenetic regulations, immunomodulation, and cell cycle regulations. SCAFs play a role in regulating apoptosis, cell cycle, and immunomodulation by inhibiting of NF‐kB and HDACs activity, DNA methylation, and regulating Akt/mTOR and MEK/ ERK signaling pathways.^9^ SCFAs such as acetate, butyrate, and propionate are synthesized by gut microbiota from fermentable non‐digestible carbohydrates. Phylum Bacteroidetes are responsible for the synthesis of acetate and propionate whereas Firmicutes are responsible for the synthesis of butyrate.^93^

**FIGURE 2:**
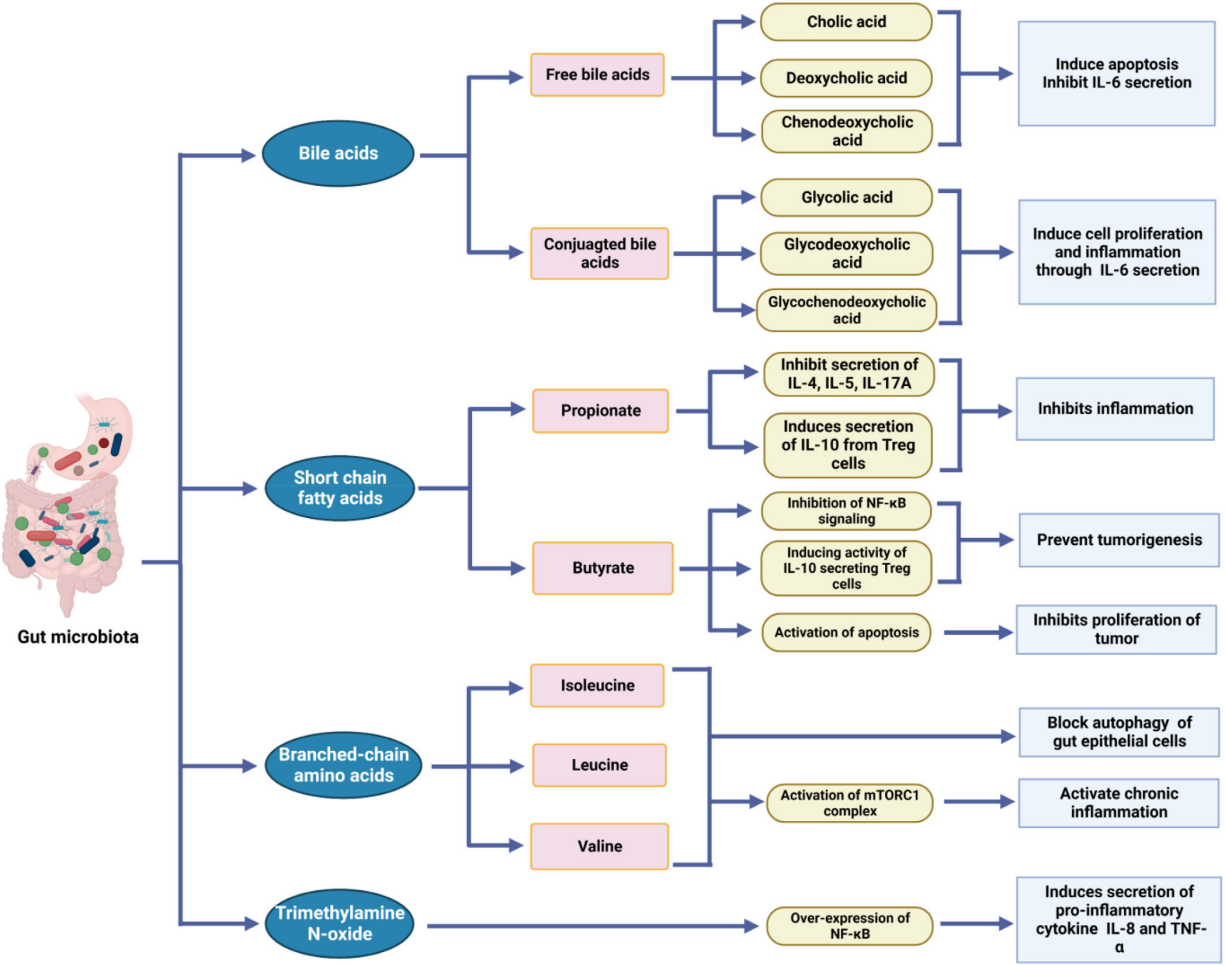
Role of gut bacterial metabolites in the development and progression of gastric cancer.

Butyrate inhibits the synthesis of TNF‐α, IL‐6, MCP‐1, iNOS, and IFN‐γ by inhibiting transcriptional activation of NF‐κB.^94^ Butyrate may prevent tumorigenesis by inhibiting the NF‐kB signaling pathway; as well as the activity of differentiated T cells that secrete IL10, and regulatory T cell. It also inhibits the proliferation of tumors through the activation of programmed cell death^95^ (Figure 2). *Faecalibacterium prausnitzii* is responsible for butyrate synthesis, which boosts the gastrointestinal immunity, and maintains the integrity of the gastrointestinal barrier. Butyrate induces the proliferation of gastric epithelial cells.^9^

Propionate inhibits the secretion of pro‐inflammatory cytokines such as IL‐4, IL‐5, and IL‐17A as well as induces the secretion of anti‐inflammatory cytokine IL‐10 from Treg cells. *H. Pylori* induce the gut microbiome to secrete BCAAs such as isoleucine, leucine, and valine which block autophagy of gut epithelial cells and activate chronic inflammation through the activation of the mTORC1 complex. TAMO induces the secretion of pro‐inflammatory cytokines such as IL‐8 and TNF‐α through over‐expression of NF‐κB^18^ (Figure 2). Polyamines (PAs) such as cadaverine, spermidine, spermine, and putrescine which are synthesized by Firmicutes in the gut are responsible for cell wall stability, siderophores formation, and antioxidant activity. *Clostridium sporogenes*, *Ruminococcus gnavus*, and *Lactobacillus* sp. are all involved in the tryptophan metabolism, which triggers tumor progression by inhibiting antitumor immune responses.^9^

A higher abundance of bacteria in the gastric fluid is responsible for the production of nitroso compounds that drive the development of gastric cancer through DNA damage. Bacteria such as *Escherichia coli*, *Streptococcus*, *Clostridium*, *Haemophilus*, *Veillonella*, *Staphylococcus*, *Neisseria*, *Nitrospirae*, and *Lactobacillus* are accountable for the development of gastric cancer. These bacteria are involved in the production of carcinogenic N‐nitroso compounds which induce over expression of proto‐oncogenes, angiogenesis, and inhibition of apoptosis.^19^ A higher abundance of nitrite has been reported in the gastric juice of gastric cancer patients.^96^ Bacterial genera *Hemophilia* and *Veillonella* are involved in the reduction of nitrate more efficiently than nitrite in the stomach.^97^ N‐nitroso compounds from tobacco smoke and diet enhance the risk of gastric cancer.^98^ Endogenous N‐nitroso compounds which are derived nitrites contribute significantly to the development of gastric cancer.^99^ *Haemophilus parainfluenzae* and *Veillonella parvula* reduced nitrate more efficiently than nitrite reduction, which escalates the concentration of nitrite in the gastric juice.^100^ Microbial metabolites may be considered as diagnostic biomarkers for GC.

A low level of gastric acid is considered to be one of the risk factors for the development of gastric cancer. Proton pump inhibitors (PPI) are responsible for reducing acid production in the gastric region. Interleukin‐1β induces gastric acid secretion, and reduced expression of IL‐1β enhances the risk of gastric cancer development.^101^ Elevation of pH induces the growth of *Lactobacillus* in the stomach which favors the growth of gastric cancer. The use of PPI treatments enhances the abundance of *Lactobacillus* and *Streptococcus*, which are responsible for the development of gastric cancer.^102^

*E coli* blocks activity of blapachone, cladribine, doxorubicin, daunorubicin, etoposide phosphate, and idarubicin. Simultaneously, it induces antitumor activity of 5‐fluorouracil (FU), 5‐fluorocytosine, mercaptopurine, and fludarabine phosphate. Cytidine deaminase of γ‐ proteobacteria reduces the toxic effect of gemcitabine through its conversion into 2′, 2′‐difluorodeoxyuridine.^103^ More research related to the role of the microbiome in improving therapeutic outcomes and reducing toxicity is required.

## 6 ROLE OF PROBIOTICS IN GASTRIC CANCER

Probiotics are living organisms that provide benefits to the host after adequate ingestion. Bacterial genera such as *Aerococcus*, *Enterococcus*, and *Lactobacillus* belong to phylum Firmicutes, and *Bifidobacterium* belongs to phylum Actinobacteria commonly use as probiotics.^104^ Antimicrobial metabolites of *L. reuteri* showed inhibitory activity against *H. pylori*.^105^ *Bifidobacterium*, and *Lactobacillus* showed antitumor activity through activation of apoptosis, and suppression of proinflammatory cytokine secretion.^9^ *Lactobacillus acidophilus* and *Lactobacillus bulgaricus* can reduce the adherence of *H. pylori* to gastric mucosal cells. *L. Bulgaricus* reduces the secretion of IL‐8 from mucosal cells through alterations of TLR4/NF‐kB pathways.^106^ *Bacillus cereus*, *Bifidobacterium infantis*, *Enterococcus faecalis*, and *L. acidophilus* synergistically suppressed inflammation in GC patients.^107^ *Lactobacillus casei*, and *Bifidobacterium* suppressed radiotherapy‐associated diarrhea in a mouse model by blocking mRNA expression of IL1b, IL6 and TNF. *Bifidobacterium pseudolongum*, *Lactobacillus johnsonii*, and *Olsenella* improved the efficacy of anti‐CTLA4 and anti–PD‐L1 immunotherapy. Higher abundance of *Bifidobacterium* in tumor microenvironment also enhanced the efficacy of anti‐CD47 immunotherapy.^108^ *Lactobacillus johnsonii*, *Lactobacillus murinus*, and *Enterococcus hirae* enhance cyclophosphamide mediated immune responses in the tumor microenvironment.^103^ *Bifidobacterium* derived hippurate blocks PD‐1 expression that drives natural killer (NK) cells to kill tumors through perforin and IFN‐γ.^109^ *Bifidobacterium pseudolongum* and *Akkermansia muciniphila secretes* inosine which reduces the tumor volume through activation of Th1 response via adenosine 2A receptors. *Lactobacillus rhamnosus* GG stimulates activity of CD8+ T cell to tumor cells through activation of dendritic cells.^110^ Probiotic strains may inhibit *H. pylori* infection by stimulating the activity of natural killer (NK) cells, and secretion of anti‐inflammatory cytokines.

## 7 CONCLUSIONS

The gut microbiome contributes to the development of tumor by altering treatment response. Age, ethnicity, diet, and gender may all have an effect on bacterial populations in the stomach and their association to cancer development. *H. pylori* are well known pathogenic bacteria that contribute to the development of GC. It is necessary to assess the functional relevance of additional microorganisms linked to progressive gastritis and stomach cancer. Exogenous lactate of LAB contributes to carcinogenesis by producing ROS, N‐nitroso compounds, and enhancing EMT. Chemotherapeutic and immunotherapeutic agents are also modulated by gut microbiota. Understanding the function of *H. Pylori* and other bacteria‐induced gastric carcinogenesis is crucial for identifying viable diagnostic and therapeutic treatments for GC. Understanding how bacteria interact with their hosts and how bacteria cause inflammatory pathways in the host is also critical for developing therapeutic targets for stomach cancer. Probiotics can be used to treat GC by preventing the growth of oncobacteria. The gut microbiota is an important determinant for making chemotherapy or immunotherapy safer and enhancing survival rates of cancer patients. Synergistic approaches such as clinical trials by using microbiota along with chemotherapy or immunotherapy are highly anticipated in the treatment of GC. Additional in vitro and in vivo experimental approaches are required to develop a better treatment plan for GC inhibition. Further research is also required to have a better knowledge of understanding microbiome‐drug interactions to improve treatment outcomes.

## AUTHOR CONTRIBUTIONS

**Indranil Chattopadhyay:** Writing and drafting entire manuscript. **Rohit Gundamaraju:** Conceptualization (equal). **Ashwin Rajeev:** Writing – review and editing (equal).

## CONFLICT OF INTEREST STATEMENT

The authors have stated explicitly that there are no conflicts of interest in connection with this article.

## ETHICS STATEMENT

Not applicable.

## Data Availability

Not available.
